# The contribution of evidence‐based practice and the practice‐based evidence approaches to contemporary Australian psychology: implications for culturally safe practice

**DOI:** 10.5694/mja2.70028

**Published:** 2025-08-14

**Authors:** Paul Gray (Wiradjuri), Dawn Darlaston‐Jones, Pat Dudgeon AM (Bardi), Kate Derry, Joanna Alexi, William Smith (Wiradjuri and Wemba Wemba), Tanja Hirvonen (Jaru and Bunuba), David Badcock, Shraddha Kashyap, Belle Selkirk (Noongar)

**Affiliations:** ^1^ Jumbunna Institute, University of Technology Sydney Sydney NSW; ^2^ University of Western Australia Perth WA; ^3^ Poche Centre for Indigenous Health University of Western Australia Perth WA; ^4^ University of Melbourne Melbourne VIC

**Keywords:** Evidence‐based medicine, Cultural competency, Research design, Psychology, Ethics, research

Psychological practice emphasises the importance of using the best available evidence to ensure accountability and promote positive outcomes for individuals and communities.^1^ These expectations are critical for community trust; however, without adequate consideration of broader processes of knowledge production, this focus can marginalise populations and perpetuate health inequities, such as those experienced by Aboriginal and Torres Strait Islander communities. Considering new professional practice expectations in psychology,^1^, ^2^ this article examines the foundations of these standards and how they might be effectively implemented. We present a conceptual exploration of empirical and constructionist perspectives on evidence and introduce guidelines for practice‐based evidence (PBE), including theoretical and practical implications to yield the best available evidence^1^ that guides culturally safe practice when working with Aboriginal and Torres Strait Islander peoples.^3^

Although the authorship team is situated within the discipline of psychology, the arguments can be extrapolated to other health disciplines. Medicine, for example, is situated within both the pure science and social science realms. Its foundation exists within the laboratory and empirical clinical trials, but it operates in the context and complexity of human patients and the cultural, historical and political milieu they inhabit. Medicine has been a pioneer in requiring students and graduates to acknowledge the diverse experiences of Aboriginal and Torres Strait Islander peoples and was among the first disciplines to include this in its curriculum. The arguments within this article enhance the foundation work provided by Indigenous leaders such as Professor Gregory Phillips.^4^

## Positionality statement

Associate Professor Paul Gray is a Wiradjuri researcher and child protection advocate, focused on promoting the safety, welfare and wellbeing of Aboriginal children, families and communities through self‐determination and reasserting Aboriginal systems and practices.

Dr Dawn Darlaston‐Jones is a non‐Indigenous woman (she/her) of British descent, who lives and works on Whadjuk Noongar Boodja as a researcher and educator. She has over 20 years’ experience in developing psychology curricula within a decolonial framework.

Professor Pat Dudgeon AM, from the Bardi people, is a psychologist, researcher and leader in Aboriginal and Torres Strait Islander mental health and wellbeing. Her area of research includes social and emotional wellbeing, Indigenous psychology, and suicide prevention.

Dr Kate Derry is cisgender (she/her), born and raised on unceded Wadjuk Noongar Boodja. She is of Burmese immigrant and Irish/English settler heritage. Her research focus on social and emotional wellbeing has challenged her to decolonise her worldview and research approach.

Dr Joanna Alexi has Cypriot heritage, was born in Larrakia Country, and is now living on Whadjuk Noongar Boodja. Dr Alexi's research has focused on decolonising psychology education and mental health systems.

William Smith (he/him) is a Wiradjuri and Wemba Wemba researcher born on Bunurong Country. His work with and for Aboriginal people focuses on embedding Aboriginal ways of knowing into psychological frameworks to support decolonisation of the discipline.

Tanja Hirvonen is a Jaru and Bunuba woman living and working on Kalkadoon country. She is a clinical psychologist in an executive position. Her interests include organisational wellbeing, social and emotional wellbeing, and decolonising psychology.

Emeritus Professor David Badcock is a seventh generation Tasmanian of English heritage. He has contributed to the design and support for education and training pathways in psychology. His research focuses on the operation of the human visual system.

Dr Shraddha Kashyap (she/her) has Indian heritage; she was born and grew up in Kenya and moved to Whadjuk Noongar Boodja 20 years ago. She is a researcher and clinical psychologist focusing on cultural safety in mental health services.

Belle Selkirk is a cisgender Noongar woman living on Wadandi Boodja. She is a clinical psychologist and researcher focusing on Indigenous psychology, decolonising psychology education and practice, and cultural safety in psychological practice.

The CONSIDER reporting criteria checklist for health research involving Indigenous peoples was completed for this article and can be found in the Supporting Information.^5^

## Expanding the definition of best available evidence

Scientific decision making should be informed by the best available evidence.^1^ The Psychology Board of Australia (PsyBA) defines evidence as “any concept, strategy, intervention or practice derived from or informed by evidence from research, including Indigenous research methodologies, that supports the quality and the relevance of a particular action or decision in a particular context for a particular use”.^2^ The forthcoming PsyBA Code of Conduct^1^ emphasises grounding practice in evidence that accounts for the social, cultural and historical contexts in which it was generated. For these changes to effectively improve outcomes, particularly for Aboriginal and Torres Strait Islander communities, psychologists require further guidance on their responsibilities in understanding how evidence is developed as well as recognising the social and political factors that shape this process, perpetuating unequal outcomes. With the PsyBA set to implement the revised Professional Competencies for Psychologists and the new Code of Conduct on 1 December 2025,^1^, ^2^ aligned with the definition and principles of cultural safety,^3^ this conversation is timely and essential. Ensuring that psychology meets the needs of Aboriginal and Torres Strait Islander peoples is critical to achieving an equitable health care system free from racism.^3^

The construction of evidence does not occur in a vacuum, and the evidence employed needs to be purpose‐specific and serve the values of those affected by these decisions.^6^, ^7^, ^8^ The examination process of evidence should recognise that the opportunity to develop and publish evidence is not equally distributed across society. The dominant research paradigm reflects the product of scientific endeavour over many generations. It reflects and entrenches social systems of power, with various groups privileged or excluded from access to resources and institutions through which knowledge and evidence may be generated.^9^ These realities must be acknowledged to ground psychology and related health disciplines on a stronger foundation of evidence. Key to this is the definition of what constitutes evidence, with empiricism and its theoretical framework of positivism being privileged to the detriment of social constructionist approaches. The former relies largely on laboratory‐based experimentation that view variables such as “culture” and “values” as something to be controlled. In contrast, interpretivist research centres these aspects of human functioning as critical to understanding lived experience. As such, this article advocates for epistemic pluralism of evidence‐based practice (EBP) that is grounded in positivism and the constructionist focus of PBE, valuing the contribution of both in the development of a more inclusive discipline to provide the “best available evidence to achieve the best possible client outcomes”.^1^

Given persistent inequities, especially affecting Aboriginal and Torres Strait Islander peoples, we recommend drawing on insights from Indigenous psychology, including both theory and practice frameworks, as a necessary step in enhancing social and emotional wellbeing. Although we acknowledge the considerable work in Australia and internationally of Indigenous psychologists to develop theoretical frameworks and practice evidence, specific examples are beyond the scope of this article. Rather, this article contributes to the conversation about the construction, development and application of evidence across health disciplines to improve individual and collective wellbeing within evolving professional practice expectations.^3^ Even though our focus is psychology, the issues discussed resonate across medicine, public health and other health professions. Therefore, a collective commitment across these disciplines, through education, research and practice, is essential to advancing equity and culturally safe care.

## Evidence and psychological practice

Responsible practice is grounded in principles of non‐maleficence and beneficence.^10^ Psychologists thus face ethical imperatives when engaged in psychological work. First, they must identify and implement the most appropriate evidence‐informed practice for individuals and communities guided by current research.^11^, ^12^ Second, psychologists must be accountable and transparent in their practice,^13^, ^14^ and third, they must engage in genuine collaborative, informed consent processes^10^ with those they serve.

These ethical imperatives enjoy broad support. However, their practical implications warrant further consideration, particularly in the context of a history of psychological practices, justified on principles of benevolence, that have demonstrably contributed to long‐lasting and even intergenerational harms, including ongoing processes of colonisation.^15^ These lived experiences emphasise the importance of evolving professional practice standards, particularly in the context of narrow social, cultural and political constructions of theory and practice.

The structural reform being implemented by PsyBA reflects these evolving standards,^1^, ^2^ extending professional practice in ways that reflect emerging discourses in critical psychological theory and practice, including commitments to Aboriginal and Torres Strait Islander health and cultural safety (eg, Code of Conduct, Principle 2; Professional Competency 8), and respectful practice with diverse peoples (eg, Code of Conduct, Principle 3; Professional Competency 7). These seek to promote safe, effective and collaborative practice that is “informed by the best available evidence to achieve the best possible client outcomes”,^1^ specifically, culturally safe practice with Aboriginal and Torres Strait Islander people and communities.^1^, ^2^, ^3^

However, if these reforms are to have a practical effect and achieve their intent of improving outcomes for clients, particularly Aboriginal and Torres Strait Islander clients, further guidance for psychologists about their responsibilities are needed. This includes building awareness of the processes by which evidence is constructed, maintained and implemented, and how the social and political components of those processes contribute to disparate outcomes.

## Evidence in practice

EBP and PBE approaches emphasise different philosophical and theoretical roots, with EBP reflecting a positivist‐based philosophy of objectivism, whereas PBE developed from a constructionist‐based philosophy of science. In psychology, it has been argued that the dominant discourse has evolved to emphasise an association with the physical sciences (ie, empiricism). This can devalue constructionist approaches and minimise consideration of social and political factors in individual and community experience, construction of meaning, and the development of knowledge.^16^, ^17^, ^18^ For example, the existing empirical evidence base in psychological research has predominantly involved Western, educated, industrialised, rich and democratic^19^, ^20^, ^21^, ^22^ populations, resulting in psychological theories and practice guidance reflecting Western cultural constructs. Consequently, certain psychological constructs or interventions may be unsuitable for diverse cohorts and contexts, contributing to inequities of outcomes experienced across populations. A broader array of evidence is needed to address these gaps and apply psychological knowledge effectively and equitably. In our view, the most productive path forward is to consider the broad array of evidence that might arise from both traditions, with consideration of the social and cultural contexts in which they apply.

We emphasise the need for an inclusive approach to defining and selecting evidence, recognising knowledge beyond the laboratory, such as cultural traditions and community‐based data. The constructionist philosophy of science acknowledges that prior knowledge and expectations affect current behaviour.^23^, ^24^, ^25^ Prior knowledge stems from individual experiences and interaction with the world.^9^, ^16^ Inclusive approaches have been applied in various psychological fields, necessitating the development of diverse research methodologies to capture the complex interplay of social, cultural, economic and political factors. These methodologies ensure that psychological practices remain relevant and effective in addressing the needs of diverse peoples, and this is increasingly reflected in global practice,^26^, ^27^, ^28^ including efforts for antiracist and decolonial practice that shift from individualist to structural perspectives.^29^

PBE recognises environmental, community, social and cultural factors influencing the perception and evaluation of information more than EBP.^8^ In alignment with National Health and Medical Research Council (NHMRC) principles, PBE allows individuals and groups to be involved in decisions that affect them, advocating for the inclusion of people with lived experience in research design.^8^ This is a critical component of ethical research with Aboriginal and Torres Strait Islander communities.^30^ Both EBP and PBE can provide meaningful insights for diverse groups through research protocols that are culturally safe and relevant. They may both provide dual paths to helpful understandings with Aboriginal and Torres Strait Islander peoples and diverse populations. We particularly emphasise a focus on Indigenous Standpoint Theory (IST) and Indigenous Knowledge Systems (IKS).^19^ Indigenous Research Methodologies (IRMs) are positioned within Indigenous standpoints and draw from IKS in the development of insights and evidence.^19^

IST examines how Aboriginal and Torres Strait Islander peoples exercise sovereignty in their ways of being, knowing and doing, and the role of power in knowledge formation.^19^ It values Aboriginal and Torres Strait Islander lived experiences, knowledge systems, politics and history, challenging Western epistemology and promoting respect for IKS.^19^ IKS enable Aboriginal and Torres Strait Islander peoples to create an evidence hierarchy for quality research, evidence, and effective intervention for social and emotional wellbeing.^8^ For example, Indigenous psychology is rooted in IST and IKS, and challenges colonial narratives and structural inequities affecting mental health.^31^ It integrates diverse approaches, blending empirical and practice‐based evidence for a broader understanding of wellbeing. Consequently, Indigenous psychology acknowledges that the unique social and political status of Indigenous peoples. The increased consideration of social and cultural factors, including engagement with specific populations and lived experience, can assist in developing a more inclusive evidence base that reflects the diversity of the Australian community.

Within the context of PBE, practice is informed by shared scientific evidence, and the contribution of experience and expertise that psychologists and health practitioners bring in their engagement with and application of the evidence base.^32^ In some areas of investigation, what constitutes the best available quality research processes and methods differ for Aboriginal and Torres Strait Islander and non‐Indigenous communities,^33^ with implications for how existing evidence is applied, including the need to avoid potential harms to Aboriginal and Torres Strait Islander peoples.^8^, ^15^ Thus, the challenge for the health disciplines, including psychology, is to provide avenues for diverse research, and practitioner education and training to ensure their knowledge encompasses relevant evidence. Ultimately, psychologists and health practitioners must recognise the necessity of gathering the best available evidence for their work, balancing the value and limitations of both EBP and PBE in the process.

## Evidence in culturally safe practice

Understanding cultural safety is central when considering EBP and PBE. Several terms are used interchangeably to describe working in a culturally safe manner, such as cultural competence, cultural responsiveness, and cultural safety.^34^ There has been a shift from cultural competency to cultural safety to achieve greater health equity and decolonise health systems.^35^ Cultural safety considers the individual, organisational, and societal steps for health equity.^35^ According to the Australian Health Practitioner Regulation Agency (Aphra):

Cultural safety is determined by Aboriginal and Torres Strait Islander individuals, families, and communities. Culturally safe practi[c]e is the ongoing critical reflection of health practitioner knowledge, skills, attitudes, practising behaviours, and power differentials in delivering safe, accessible, and responsive healthcare free of racism.^3^

Understanding and applying cultural safety is embedded throughout PsyBA's Professional Competencies for Psychologists and Code of Conduct as a core expectation of professional practice. PsyBA mandates that psychology registrars demonstrate an understanding of how to work with people from diverse cultural backgrounds, and supervisors approved by the PsyBA are required to be culturally responsive in supervision.^36^ These considerations also apply to conducting research with Aboriginal and Torres Strait Islander individuals and communities. Even with cultural safety operationalised at the health practitioner level, cultural safety must be embedded within the system itself, including policies, laws and programs that systematically address structural racism.

As outlined in the Professional Competencies,^2^ psychologists must employ ongoing critical self‐reflexivity, not just in their practice but also concerning the knowledge base of the discipline and the methods employed to generate that evidence to determine the appropriateness of that evidence. PsyBA explain:

Culturally safe practice is the ongoing critical reflexivity of health practitioner knowledges, skills, attitudes, actions, practising behaviours and power differentials in delivering safe, accessible and responsive healthcare free of racism and discrimination. It includes, but is not limited to, the history, spirituality and relationship to land, and other cultural and social determinants of health and wellbeing in Aboriginal and Torres Strait Islander communities.^2^

Therefore, psychologists must be aware of their own cultural identity and positionality concerning the persons, groups and communities with whom they are working. The application of critical self‐reflexivity can facilitate processes of cultural safety and health equity when selecting best available evidence to support working with Aboriginal and Torres Strait Islander peoples.

## Understanding the characteristics of those receiving psychological services

When deciding on a course of action in psychological or health care practice, implementing research evidence requires a judgement as to its applicability to the unique characteristics and circumstances of those receiving services.^37^ Characteristics of the client, group and/or organisation, therefore, are important contextualising factors that need to be carefully considered.^38^ For example, in clinical contexts, client attributes include individual variations of the presenting issue, needs, history of treatment response, motivation for change, values, culture, language proficiency, and personal preferences.^37^, ^38^, ^39^, ^40^, ^41^ In implementing an intervention, tailoring to the client's characteristics (eg, the literacy level of materials) can often be implemented to enhance treatment applicability and acceptability without undermining the fidelity of the core treatment elements that make treatment effective.^39^ Research has highlighted the importance of shared decision making in the health care delivery process and the value of client input in selecting a preferred treatment approach. Engaging clients in decision making promotes an effective client–practitioner relationship and is associated with improved outcomes and decreased risk of dropout.^40^, ^41^ This may similarly operate at the level of communities, providing input into the design of available approaches and the application of evidence. In all research, education and policy contexts, the historical, political, economic, social and cultural determinants of health are relevant, as they often place boundaries on the range of intervention options that may be considered.

There are many factors for psychologists and health practitioners to consider when determining the likely efficacy of an intervention. For instance, if the concept of mental health differs among groups of people being provided with the same intervention, then measuring effectiveness (and efficacy) for all groups in the same way may not yield accurate information for all groups. Consequently, using Western definitions of mental health and research methodologies to determine the effectiveness of an intervention for Aboriginal and Torres Strait Islander peoples might be undermined by misalignment with Aboriginal and Torres Strait Islander perspectives that consider interrelationships between physical and mental health and individual and collective health.^42^ This holistic concept of health is described as social and emotional wellbeing.^42^ To measure the effectiveness and efficacy of an intervention aimed at improving the social and emotional wellbeing for Aboriginal and Torres Strait Islander peoples, for example, culturally appropriate methodologies and measures are imperative.^43^ It should be noted that practical applications of social and emotional wellbeing can apply to other groups beyond Aboriginal and Torres Strait Islander peoples. For instance, collective influence, including the social determinants of health such as poverty, exclusion, underemployment, and access to resources, each affects the wellbeing of individuals, families and communities regardless of ethnicity. Scientists in the areas of environmental, climate and biodiversity fields are seeking Indigenous knowledge; psychological scientists should similarly consider the beneficial outcomes that can be achieved by utilising Indigenous knowledges and its applicability to other cultural groups.

There is, however, a lack of adequate evidence demonstrating that Western models of practice are meaningful, valid or effective with Aboriginal and Torres Strait Islander peoples^44^ or applicable with other culturally diverse communities.^45^ Western therapeutic approaches are less effective and can indeed be detrimental for Aboriginal and Torres Strait Islander peoples if not appropriately situated within Indigenous knowledges, cultures, and lived experiences.^46^ Rather than relying on Western models of health and wellbeing, constructions of internal mental states and experiences, and associated diagnostics and responses, culturally safe research that adheres to Indigenous definitions of quality is needed.^33^ A practice approach that prioritises evidence without considering these issues entrenches, rather than alleviates, inequality and injustice and must be avoided.

## Implementation principles of practice‐based evidence

A strength of PBE approaches is the opportunity to consider the diverse perspectives and experiences of individuals and groups. This is particularly relevant to underserved populations who have been silenced through the development of EBP and continue to experience inequitable outcomes from the application of interventions. Given the need to address these persistent inequities, we offer the following principles to implement PBE approaches, which have been informed by the NHMRC ethical guidelines for research with Aboriginal and Torres Strait Islander peoples,^47^ the Cultural Respect Framework,^34^ and the Aboriginal and Torres Strait Islander Quality Appraisal Tool,^33^ and include specific prompts for application with Aboriginal and Torres Strait Islander peoples as an example. We offer insights into PBE approach in the Box.

Box 1 **Insights into practice‐based evidence (PBE) approach**

**Table:** 

The PBE approach includes: Understanding that PBE is local, community‐led, and does not have to be generalisable to other settings. Recognise the diversity of Aboriginal and Torres Strait Islander lived experiences and knowledges, and the epistemological equivalence these knowledges hold. Take time to explore the cultural context of the person or group, ensuring that Aboriginal and Torres Strait Islander beliefs, values, knowledge systems, practices and communication styles, including regional variations, are prioritised and appropriately incorporated. Develop ongoing reciprocal relationships with communities served, such as Aboriginal and Torres Strait Islander community groups, Aboriginal Community Controlled Health Organisations, Elders, cultural advisors, and Traditional Healers. For examples of best‐informed practice, review both academic and grey literature, and community reports that are specifically led by people from the cultural group. Carefully review publications to determine if they have been led by Aboriginal and Torres Strait Islander peoples. Engage in ongoing self‐reflexive practice in cultural safety. Review cultural safety at organisational levels, including addressing power differentials to elevate structurally oppressed Aboriginal and Torres Strait Islander peoples and voices.

## The benefits and implementation of practice‐based evidence

From a constructionist perspective, the role of language and narrative, how people make sense of their experiences, and the complex interactions that derive from their experiences are all central to understanding the person, family and community. The inclusion of this broader range of evidence is desirable in all contexts, including when devising an EBP procedure. This section briefly discusses how IST and IKS are central to understanding the experiences of Aboriginal and Torres Strait Islander peoples within historical, political, social, and cultural determinants of health. Both IST and constructionist approaches emphasise the importance of reflexive practice and a relational process that is cyclical and ongoing. The Supporting Information (section A) presents a fictional example of Aboriginal and Torres Strait Islander lived experience to illustrate the complex, cyclical and reflexive method of enquiry and knowledge gathering inherent in PBE.

PBE is an approach situated within IKS and IST, which contrasts with the dominant perspective of EBP. This approach to evidence is focused on the specific context of its application. Although there might be some commonalities that can transfer to other settings, this is not a requirement. What works for the person or group at a local specific level is right for that person or group at that location. This approach raises the issue of power differentials in therapeutic, employment and educational settings. The pervasive dominance of Western norms and practices effectively silences other experiences and ways of knowing, resulting in a form of professional arrogance that assumes that one knowledge system is superior to all other forms of knowing. Further, this professional arrogance can deter people from seeking, accessing, and complying with health and mental health services in the future. Ongoing self‐reflexive practice is important, but applying reflexivity to one's disciplinary knowledge systems is equally critical.

There are several barriers that prevent EBP and PBE from being optimally applied. At the individual level, a significant barrier is the lack of training and skills in constructionist‐ and subjectivist‐based methodologies, the application of ongoing critical reflexivity on the evidence hierarchy and the importance of context, and knowledge of culturally safe practices. Barriers at the organisational level include, but are not limited to, time constraints, resource limitations, and an environment that prevents ongoing critical reflexivity and culturally safe practices. By addressing these barriers through targeted strategies, individuals and organisations can enhance the implementation of evidence in their practice and contribute to the development and application of evidence‐informed approaches for diverse populations, addressing persistent inequities, and ultimately improving the quality of care provided. As a brief example, psychologists and health practitioners should seek information from the academic literature while being cognisant that many academic journals use criteria that have led to it being difficult to publish IKS research. Moreover, practitioners need to be cognisant of carefully reviewing the authorship of academic literature to ensure it includes Aboriginal and Torres Strait Islander authors, meaningful partnership with Aboriginal and Torres Strait Islander communities and organisations, or the use of Aboriginal and Torres Strait Islander‐developed screening tools. Where there are identified gaps, one would need to examine the broader literature sources, including community‐based texts, that are more likely to report diverse approaches. Finally, practitioners should seek cultural mentorship and advice from Aboriginal and Torres Strait Islander peers, colleagues and communities. The Supporting Information (section B) provides further examples of ways to address some of the barriers noted in this article.

## Future directions

Changes to the PsyBA standards are a positive step in psychology but must be matched by significant action to ensure that implementation rises beyond the facade of practice improvement and into the lived experiences of communities, including Aboriginal and Torres Strait Islander peoples, in particular. Administrators and regulatory bodies should partner with Aboriginal and Torres Strait Islander communities in developing guidance and oversight for evidence‐informed practice. This should include regulation of practice standards and their implications for Aboriginal and Torres Strait Islander communities by, for and of Aboriginal and Torres Strait Islander peoples, rather than retaining this in the hands of non‐Indigenous professional associations and regulatory bodies. Further, there must be significant investment in Aboriginal and Torres Strait Islander theory and practice development, through research processes that adhere to Indigenous research ethics and place Aboriginal and Torres Strait Islander communities at the centre of research efforts, to address the longstanding marginalisation of these perspectives in the discipline and associated evidence base. Consistent with these ethical frameworks, this research must ensure Indigenous cultural and intellectual property is retained by Aboriginal and Torres Strait Islander peoples and is subject to Aboriginal and Torres Strait Islander legal frameworks of knowledge custodianship, and not inappropriately dispossessed or privatised by researchers or non‐Indigenous research institutions where benefits for Aboriginal and Torres Strait Islander people might not be effectively realised.

## Conclusion

This article has examined the value of both EBP and PBE and the application of each of these approaches. Both approaches have unique benefits, and each has a useful role to play in the practice and education, and training of psychologists and health practitioners. PsyBA's Professional Competencies for Psychologists and Code of Conduct represent an important shift toward recognising the importance of cultural context, lived experience, and IKS in shaping what constitutes the best available evidence. These developments offer important insights for health disciplines, including medicine, which similarly straddles scientific and human‐centred domains. The necessity to move toward epistemic pluralism, cultural safety, and critical reflexivity highlights the need for all health disciplines to embrace both rigorous scientific evidence and community‐led, contextually grounded knowledge. Adopting a broader and more inclusive approach to evidence represents an important step toward addressing the persistent inequities experienced by many Aboriginal and Torres Strait Islander peoples and diverse communities.

## Open access

Open access publishing facilitated by The University of Western Australia, as part of the Wiley ‐ The University of Western Australia agreement via the Council of Australian University Librarians.

## Competing interests

Pat Dudgeon AM is a Guest Editor for the 2025 NAIDOC Week *MJA* Special Issue and was not involved in any editorial decision making about this article.

## Provenance

Not commissioned; externally peer reviewed.

## Author contributions

Gray P: Conceptualization, methodology, resources, supervision, writing – original draft, writing – review and editing. Darlaston‐Jones D: Conceptualization, methodology, resources, writing – original draft, writing – review and editing. Dudgeon P: Conceptualization, methodology, resources, supervision, writing – original draft. Derry K: Conceptualization, methodology, project administration, resources, writing – original draft. Alexi J: Conceptualization, methodology, project administration, resources, writing – original draft. Smith W: Writing – original draft, writing – review and editing. Hirvonen T: Conceptualization, methodology, resources, writing – original draft, writing – review and editing. Badcock D: Conceptualization, methodology, resources, writing – original draft. Kashyap S: Conceptualization, methodology, resources, writing – original draft. Selkirk B: Conceptualization, methodology, project administration, resources, supervision, writing – original draft, writing – review and editing.

## Supporting information


Supplementary examples
