# Supplementary material for: The contribution of evidence‐based practice and the practice‐based evidence approaches to contemporary Australian psychology: implications for culturally safe practice
Source: Med J Aust. 2025 Aug 14;223(6):282–8. doi: 10.5694/mja2.70028 (PMC12434551; doi:10.5694/mja2.70028)
Supplement: Supplementary file 1 — Supplementary examples [file MJA2-223-282-s001.pdf]

Supporting Information

Supplementary material

This appendix was part of the submitted manuscript and has been peer reviewed.
It is posted as supplied by the authors.

Appendix to: Gray (Wiradjuri) P, Darlaston-Jones D, Dudgeon AM (Bardi) P, et al. The contribution of evidence-based practice and the practice-based evidence approaches to contemporary Australian psychology: implications for culturally safe practice. *Med J Aust* 2025; doi: 10.5694/mja2.70028.

CONSIDER Statement

The contribution of evidence-based practice and the practice-based evidence approaches to contemporary Australian psychology: Implications for culturally safe practice.

**Governance**
Partnership: The original project was a collaboration between the Australian Indigenous Psychological Education Program (AIPEP), Transforming Indigenous Mental Health & Wellbeing (TIMHWB), Australian Indigenous Psychologists Association (AIPA), and the Australian Psychological Society (APS), formalised by representatives of each organisation participating in a Working Group. The members of the original Working Group contributed to the development of an initial extended manuscript. The APS later withdrew from the collaboration. The remaining members worked in partnership to make significant changes to the original manuscript. Therefore, the current paper represents the Intellectual Property (IP) of the current authors.<br><br>Accountability: The current paper was led by three senior Indigenous psychology researchers (Associate Professor Paul Gray, Professor Pat Dudgeon, and Belle Selkirk). The Indigenous (Professor Paul Gray, Professor Pat Dudgeon, Belle Selkirk, Will Smith, Tanja Hirvonen) and non-Indigenous (Dr Dawn Darlaston-Jones, Dr Joanna Alexi, Dr Kate Derry, Dr Shraddha Kashyap, Emeritus Professor David Badcock) authors are committed to working in authentic partnership and to being guided by Indigenous governance protocols. The project had a risk management plan which included the importance of engaging with Indigenous stakeholders.<br><br>Intellectual property: All members of the original Working Group contributed to the development of an initial extended manuscript. The Working Group members representing the APS withdrew from the collaboration, including authorship, and have not contributed to the current version of the manuscript. This version of the paper has been substantially revised and is the original work and intellectual property of the current authors.
**Prioritization**
The collaboration was developed in response to the aims and mission of AIPA, AIPEP and TIMHWB.
**Relationships (Indigenous stakeholders/participants and Research Team**

Ethical Guidelines, processes, and approvals: This discussion paper did not require formal research ethics but was guided by the six values of the 2018 National Health and Medical Research Council (NHMRC) Ethical in research with Aboriginal and Torres Strait Islander Peoples and communities: Guidelines for researchers and stakeholders.<br><br>Stakeholder Involvement: This project involved extensive iterative yarns between representatives of each organisations (TIMHWB, AIPEP, AIPA, and APS) in a Working Group. The membership of the Working Group changed over time, with the current authors representing the Working Group that undertook the major revision of the manuscript. The major functions of the Working Group were to:<br><br>• Provide a critical and cultural review of research activities and implementation and ensure the project was grounded and relevant.<br>• Assist in providing feedback and insights on the manuscript.<br>• Develop and facilitate a communication strategy for dissemination.<br><br>Expertise: The Working Group consists of a collective of Indigenous and non-Indigenous clinicians and researchers with diverse levels and areas of expertise and training. The relationship between Professor Dudgeon and each organisation (TIMHWB, AIPEP, AIPA, and APS) has been nurtured for many years, and this project represented another step forward in their commitment to cultural responsiveness and safety within their organisational culture and membership.
**Methodologies**
Methodological approach: Qualitative components included iterative yarning among the authors, which included Indigenous and non-Indigenous health care professionals from each organisation.<br><br>Consideration of physical, social, economic, and cultural environments: The paper reflects the importance of considering broader systems and structures in psychological practice. Leadership of Indigenous Group members ensured that all members were engaged in a culturally safe and respectful manner.
**Participation**
Consent: The Indigenous (Associate Professor Paul Gray, Professor Pat Dudgeon, Belle Selkirk, Will Smith, Tanja Hirvonen) and non-Indigenous (Dr Dawn Darlaston-Jones, Dr Joanna Alexi, Dr Kate Derry, Dr Shraddha Kashyap, Emeritus Professor David Badcock) authors of the current paper have consented to publication.

Resource demands: Working Group members from TIMHWB, AIPEP, and AIPA convened in October 2024 to resubmit a substantially revised version of the manuscript in January 2025. No one was paid for their involvement beyond the organisations willingness to contribute staff time towards these activities.<br><br>Samples: No biological tissues or samples were used in this project.
**Capacity**
Indigenous research capacity: The Working Group meetings provided opportunities for two-way learning and supported the leadership and research capacity of Aboriginal Working Group members.<br><br>Professional development: Aboriginal clinicians and researchers provided mentoring and capacity building to one another. Through regular meetings involving participation, collaboration, and education, the project team fostered an ongoing, mutually beneficial, and respectful relationship that facilitated two-way learning and knowledge sharing between the members.
**Analysis and interpretation**
The analysis was overseen by three senior Indigenous psychology researchers (Associate Professor Paul Gray, Professor Pat Dudgeon, and Belle Selkirk), and endorsed by the Working Group.
**Dissemination**
Dissemination: The dissemination of this work, once published, will occur through each of the organisations involved, their partners, and members or networks. This work is of critical importance in supporting the implementation of the upcoming changes with Psychology Board of Australia.<br><br>Processes of knowledge translation and implementation: This article, and the findings and learnings from this project will be used to support the work of AIPEP, TIMHWB and AIPA; as well as support the psychology profession in meeting the upcoming changes with Psychology Board of Australia to continue to their commitment to cultural safety and reconciliation.

Section A

The benefits and implementation of practice-based evidence

From a constructionist perspective, the role of language and narrative, how people make sense of their experiences, and the complex interactions that derive from their experience, are all central to understanding the person, family, and community. The inclusion of this broader range of evidence is desirable in all contexts, including when devising an evidence-based practice (EBP) procedure. This section discusses Indigenous Standpoint Theory (IST) and Indigenous Knowledge Systems (IKS), and how these are central to understanding the experiences of Aboriginal and Torres Strait Islander peoples within broader ecological factors (i.e., historical, political, social, and cultural determinants of health). Both IST and constructionist approaches emphasise the importance of reflexive practice and a relational process that is cyclical in nature. The Supplementary Material A document presents a fictional example of Aboriginal and Torres Strait Islander lived experience to illustrate the complex, cyclical, and reflexive method of enquiry and knowledge gathering inherent in practice-based evidence (PBE). It is important to note that this fictional example is a representation of lived experiences, and it is not based on a single person, family or community's lived experience.

Consider the following practice example:

Aaron is an Aboriginal man who lives with his wife and children in an urban Australian city. He has several siblings who also have children. He is considered another father and caregiver to his brothers' children, who also call him Dad. Sadly, one of his nephews, whom he called son, died suddenly. Aaron grieves deeply for his nephew. So do his brother and sister-in-law and their children, as well as his grandparents, other aunts/uncles, and sister-cousins and brother-cousins. The family made the decision to take their loved one's body to a specific area on Country 30 kilometres outside an urban centre, where extended family members in their kinship system would join them to participate in mourning/ceremony/sorry time. This is very important for Aaron and his family. Culturally, Aaron has specific roles he is required to complete as part of the ceremony. Unfortunately, because of a COVID-19 community outbreak, restrictions were put in place preventing people from travelling freely between and throughout the area and the state. This means that Aaron and his immediate family cannot travel to the area on Country as planned, even though some extended family members are able to do so. Aaron and

his family are understandably conflicted and heartbroken. Some Elders expressed concerns about the wellbeing of their loved ones' spirit if cultural protocols cannot be performed in the right way. Some Elders recalled other upsetting times in their life when they were prevented from being on Country. Some family members expressed worries about Elders and physically vulnerable family members being put at risk of contracting the virus if they travelled or attended gatherings. Some family members expressed sadness and anger that they were not able to attend the ceremony. Some family members felt numb and disconnected. Aaron described feeling a strong sadness in his heart, as well as having difficulty getting out of bed and going to work. He also reports hearing his nephew's voice in the evenings and has vivid images of him in his dreams. Aaron reaches out to the local community mental health clinic for support.

Sarah is a registered psychologist and is employed in the community mental health clinic. She has European ancestry. She has been practising for 5 years and has received limited cultural awareness and cultural safety education during her university training. She presents Aaron's referral in clinical peer supervision for discussion and feedback regarding ways to engage with Aaron that are culturally appropriate. The team's clinical supervisor recommends an internal referral to the team's psychiatrist for diagnostic clarification and treatment recommendations as a crucial first step. Another psychologist recommends connecting with the local Aboriginal Community Controlled Health Service to see if they offer any grief services or resources. Sarah has already sought guidance from the academic literature on Aboriginal and Torres Strait Islander grief responses and is challenged by the limited amount of qualitative and quantitative research available and even less that is authored by Aboriginal and Torres Strait Islander people. Sarah finds a number of theoretical discussion papers and opinion pieces, especially those written by Aboriginal and Torres Strait Islander people, which provide important guidance in exploring the literature.

Practice-based evidence implementation approach informed by IST and IKS:

The IKS approach places the cultural context of the person/group at the centre of considerations. In this context, it means positioning the beliefs, values, and knowledge systems of Aboriginal and Torres Strait Islander peoples at the forefront. The Australian Health Practitioner Regulation Agency (Ahpra) and its 15 National Boards have made formal commitments to embed cultural safety and anti-racism across all Ahpra regulated professions

actively working towards a healthcare system free from racism with a specific focus on Aboriginal and Torres Strait Islander peoples. These policy changes challenge the one-size-fits-all approach and promote organisations, new graduates, and existing health professionals in all Ahpra regulated professions to think more critically and reflexively about ways of working with Aboriginal and Torres Strait Islander peoples. This may include organisations and individual practitioners engaging in cultural safety training, developing long standing relationships with Aboriginal and Torres Strait Islander governed organisations (e.g., Aboriginal Community Controlled Health Services), and critically examining the cultural safety of the organisational policies and processes that may discriminate, prejudice, or mislabel Aboriginal and Torres Strait Islander peoples.

In addition to Sarah's own self-reflexive process on how to provide culturally safe care for Aaron and his family, the organisation in which she works should provide support and opportunities for meaningful partnerships with the Aboriginal and Torres Strait Islander community, specifically engaging with and learning from Elders, Traditional Healers, and cultural advisors. In parallel with this, individuals and organisations should draw on the wealth of written works from Aboriginal and Torres Strait Islander leaders, researchers, and scholars. Both oral and written IKS have value and provide important insights into effective ways of working with Aboriginal and Torres Strait Islander peoples, which Western empirical evidence may lack.

In adopting an IKS approach, Sarah recognises the centrality of relationships and the importance of allowing time to build a genuine connection with Aaron and his family. Listening deeply without judgment and allowing for silence is important for Aboriginal and Torres Strait Islander peoples. Sarah should shift her communication style by slowing down in the amount and frequency of assessment questions asked and allowing Aaron's personal narrative as well as his family's collective narrative to unfold. Yarning is a culturally validated methodology and narrative discourse for Aboriginal and Torres Strait Islander peoples. For example, the IKS approach would ask "What has been happening in your community?", rather than "What are your symptoms?". Organisational systems might recognise the importance of time for Aaron and his family by allowing for flexible assessment processes and flexibility in the scheduling of appointments, for example.

The IKS approach honours Aboriginal and Torres Strait Islander beliefs and values by considering the interconnectedness between person, family, community, and culture. It is important to consider the broader ecological and historical context for Aboriginal and Torres Strait Islander people's wellbeing. Sarah should consider what other losses or trauma are being experienced and compounded for Aaron, his family, and his community, and how government regulations are impacting the grief process, family functioning, and the ability to engage in necessary cultural protocols. Particular attention should be paid to the lived experiences of so called "Aboriginal protection policies" and other historical and contemporary forms of government control imposed since the colonisation of Australia, including the widespread dispossession of land and the spread of disease, massacres, cultural genocide, forced child removals and lack of truth telling, resulting in transgenerational experiences of trauma, loss and powerlessness among Aboriginal and Torres Strait Islander families and communities.

The IKS approach values cultural explanations of grief and loss, which is critical in Sarah's understanding of Aaron's personal experience. There are different presentations of hearing and seeing associated with grief in Aboriginal and Torres Strait Islander cultures. Aaron's experience of hearing his nephew's voice may be a cultural phenomenon and consultation with an Elder, Traditional Healer or cultural advisor prior to any Western psychological or psychiatric intervention is appropriate. Without considering the cultural context and connecting with community, the conceptualisation and intervention could be quite different and potentially harmful. Seeking cultural supervision will help Sarah develop culturally-informed and trauma-informed conceptualisations. A collaborative assessment (rather than one done in isolation) with Aaron, his family, community members, and/or Elders will help to minimise Sarah's cultural biases and constructively work towards developing a culturally responsive safety care plan. Sarah should observe and respect cultural protocols regarding ways of talking about the deceased person, using their name, or other norms as identified by an Elder, Traditional Healer or cultural advisor. She should seek clarification about what is and what is not appropriate and seek permission about whether it is okay or not okay to talk about certain issues.

The IKS approach prioritises Aboriginal and Torres Strait Islander holistic beliefs of health and wellbeing. This may require Sarah to be open and accepting in exploring topics that may not at first seem directly related to the referral reason of grief and loss, or may involve others in the family. For instance, showing interest in the family functioning and how the

children in the family are being supported at school. For many Aboriginal and Torres Strait Islander peoples, recovery is likely to take the form of healing in the community, rather than interventions in the treatment room. Healing for Aboriginal and Torres Strait Islander peoples includes participating in ceremony, spending time with Elders or Traditional Healers, taking traditional medicines, participating in yarning circles, engaging in art, song, dance, and storytelling, being on Country and caring for Country, and engaging in programs aimed at boosting empowerment and self-determination. The IKS approach encourages cultural continuity and self-determination, such as the collaborative decision making between Aaron, his family, Elders, and the Traditional Healer regarding what healing practices need to be undertaken, including what ceremony or cultural protocols are possible given the circumstances, so that the grief process can be performed in a culturally appropriate way.

Such an approach, situated within IKS and IST is PBE, which contrasts with the dominant perspective of EBP. PBE here is local, community-led, and does not have to be generalisable to other settings. While there might be some commonalities which can transfer to other settings, this is not a requirement. What works for the person/group at a local specific level is right for that person/group at that location.

Culturally safe care also needs to be reflected in the organisational policies of Sarah's workplace. A policy stipulating EBP that fails to acknowledge and systematically excludes consideration of cultural diversity and culturally centred care is likely to be harmful to Aaron and his family. Sarah's colleague suggested reaching out to the local Aboriginal Community Controlled Health Organisation, which demonstrates that flexible and culturally safe approaches are encouraged. Organisational culture is also relevant to Aaron's employer. Employing an IKS approach, Sarah would ask Aaron if his workplace has provisions for cultural leave and would suggest steps for him to find and apply for such leave.

Similarly, she would enquire about the support available for the children from their schools. A Western view would see the schools offering individual counselling support for the children from a school psychologist, chaplain or counsellor. Schools should strive to establish relationships with the local community and Elders, allowing them to incorporate appropriate protocols into their policies and practices, thereby fostering cultural safety. This would also include the school sharing information with Aaron about his nieces and nephews and providing a

family-based support framework.

This approach also raises the issue of power differentials in therapeutic, employment, and educational settings. The pervasive dominance of Western norms and practices effectively silences other experiences and ways of knowing, resulting in a form of professional arrogance that assumes that one knowledge system is superior to all other forms of knowing. Further, this professional arrogance can deter people from seeking and accessing health and mental health services in the future. Self-reflexive practice is important, but applying reflexivity to one's disciplinary knowledge systems is equally critical.

Sarah would also seek information from the academic literature, but would also be cognisant of the fact that many academic journals use criteria that have led to it being difficult to publish IKS research. She also needs to be cognisant of carefully reviewing the authorship of academic literature to ensure it includes Aboriginal and Torres Strait Islander authors or Aboriginal and Torres Strait Islander developed screening tools. Where there are identified gaps, she would need to examine the broader literature sources, including community-based texts, that are more likely to report diverse approaches. She would also seek cultural mentorship and advice from community, Aboriginal peers, colleagues, and communities.

Section B

Identifying and addressing barriers to implementing PBE and EBP

There are several barriers at the individual practitioner level and the organisational level that prevent EBP and PBE being optimally applied. A better understanding of these barriers can provide practitioners with the best opportunity to maintain integrity with whichever approach they are applying, and to ensure the best outcomes for those they work with.

Barriers at the practitioner level

A significant barrier to implementation for many practitioners is a lack of training and skills in the constructionist- and subjectivist-based methodologies. Addressing this barrier requires further demonstration of the efficacy of such methods and in establishing when they are best used and may require advocating for broadening accreditation standards, as well as targeted recruitment of academics with competence in both approaches, collaboration between disciplines, mentoring, workshops and the use of appropriate textbooks. Barriers at the practitioner level include:

- Lack of time and resources to learn/engage in a range of methods of enquiry.
- Lack of knowledge and skills in implementing EBP and PBE, including critical reflection of the evidence hierarchy and the importance of context and how to evaluate the efficacy of treatments.
- Valuing common therapeutic factors over interventions based on prior evidence of efficacy for presentations.
- Failure to consider intuition based on relevant experience in tandem with evidence-based decision making.
- Failure to carefully analyse whether the research evidence, however acquired, is pertinent to the target group. Over-reliance on sourcing information from empirical literature and limited awareness of culturally appropriate resources, for instance, the grey literature and PBE exemplars, for example, from Aboriginal and Torres Strait Islander community groups, Elders, Traditional Healers, and cultural advisors.
- Awareness that the research context can inappropriately favour certain treatment modalities over others.
- An unnecessarily narrow view of the types of evidence that may be useful.

- Limited exposure to culturally responsive practices.
- Limited ongoing critical self-reflexivity.

These barriers can be addressed by providing continuing education opportunities, evaluation of efficacy where needed and advocacy to encourage the following strategies, attitudes, and practices among practitioners. Strategies beneficial for all practitioners include:

- Being an active and wide consumer of the scientific research literature, and being sensitive to implicit biases.
- Being familiar with research terminology and the strengths of different research designs.
- Being active in monitoring practice outcomes and determining whether the chosen interventions are having the desired results or whether a different course of action is warranted.
- Engaging in supervision and continuing professional development.
- Engaging in critical thinking and reflexive practice to ensure quality decision making.
- Emphasising the importance of therapeutic relationships and recognising different standpoints and positionalities.

Barriers at the organisational level

At the organisational level, barriers include the time constraints experienced by busy practitioners, difficulty accessing high-quality training or literature, organisational culture and attitudes towards EBP and PBE. These factors can all contribute to a drift from practice based on evidence and an erosion in the quality of care provided.

Lack of time and resources

Practitioners often lack ready access to resources, such as library facilities, online databases, contemporary textbooks, and manuals, as well as access to training and supervision. When they do have access to such resources, they can be faced with an overwhelming amount of research literature, taxing the decision-making process and making judgments about which intervention to use, difficult. Some disadvantages of a rigorous decision-making process include the time and commitment required of already time-poor practitioners. Organisations also face demands, including costs and other resources required to provide training and supervision to staff to improve and maintain their skills. The barriers are best approached by collaborative efforts to address the available evidence. These may be continuing education sessions, peer groups that distribute the reading or consultation efforts and, ideally, allocated time to allow for ensuring the

currency of knowledge. The solutions will necessarily vary from workplace to workplace, but best practice can only arise from a comprehensive knowledge base and so this barrier needs to be addressed.

Workplace attitude and culture

Culture and climate within organisations may have a direct bearing on the adoption and adherence to evidence-based or practice-based decisions amongst staff employed within organisations. Higher levels of organisational support for EBP have been shown to be associated with more positive staff attitudes towards the adoption of EBP, translating into actual adoption of EBP. This suggests that workplace culture is likely to be an important factor in adopting a more balanced approach to the use of EBP and PBE in forming the evidence base.

Key elements at the organisational level which help address these barriers and thus support the adoption of both EBP and PBE include:

- Having leadership that promotes the merits of both approaches.
- Building capacity to engage in practice that is informed by both strategies.
- Having an effective implementation framework and the infrastructure to support and maintain a culture inclusive of both strategies.

Specifically, this involves:

- Provision of strong leadership which communicates a vision across the organisation.
- Fostering a workplace culture which values, supports and embeds appropriate processes in everyday practice.
- Consideration of the use of specialist staff with expertise within the organisation as needed.
- Providing access to training and other regular professional development activities.
- Providing access to resources such as computers, databases, and other user-friendly research dissemination resources.
- Synthesising and disseminating relevant research evidence to staff.
- Addressing the time constraints experienced by staff and ensuring adequate time for critical thinking and reflexive processes.
- Providing access to supportive supervision which uses a critical approach.

- Consideration of the use of “communities of practice”, which bring together various engagement strategies and practitioners, to share experiences and practice knowledge.
